# Infections caused by clonal spread of metallo-beta-lactamase-producing *Enterobacter cloacae* complex isolates at a southern Taiwan hospital

**DOI:** 10.1128/spectrum.00234-25

**Published:** 2025-06-10

**Authors:** Cong-Tat Cia, Shu-Li Su, Pei-Fang Tsai, Yu-Cheng Su, Nan-Yao Lee, Wen-Chien Ko, Po-Lin Chen

**Affiliations:** 1 Department of Internal Medicine, College of Medicine, National Cheng Kung University, National Cheng Kung University Hospital 639603, Tainan, Taiwan; 2 Center for Infection Control, College of Medicine, National Cheng Kung University Hospital, National Cheng Kung University 38026, Tainan, Taiwan; 3 Department of Pathology, National Cheng Kung University, College of Medicine, National Cheng Kung University Hospital 568614, Tainan, Taiwan; 4 Institute of Basic Medical Sciences, College of Medicine, National Cheng Kung University 34912, Tainan, Taiwan; 5 Department of Medicine, College of Medicine, National Cheng Kung University 665116, Tainan, Taiwan; Taichung Veterans General Hospital, Taichung, Taiwan

**Keywords:** carbapenem-resistant Enterobacterales, carbapenemases, *Enterobacter cloacae* complex, Taiwan

## Abstract

Carbapenem-resistant *Enterobacter cloacae* complex (ECC) has become a clinically important pathogen with increased incidence worldwide in recent years. In Taiwan, IMP-8 accounted for most carbapenemase produced by ECC in earlier studies. Clinical ECC isolates resistant to ertapenem, imipenem, and meropenem at the study hospital between 2014 and 2022 were collected at a medical center in southern Taiwan. The isolates were subjected to multilocus sequence typing, species identification by *hsp60* sequencing, polymerase chain reaction detection of beta-lactamase genes, antimicrobial susceptibility tests, and whole genome sequencing analysis. Fifty-four ECC isolates causing infections were included. Bloodstream (20 episodes, 37.0%) and lower respiratory tract infections (14, 25.9%) were the common infection types. The crude in-hospital mortality was 51.8%. Carbapenemase genes were detected among 90.7% of the isolates, and *bla*_IMP-8_ remained to be the most common gene. The amikacin and colistin resistance rates were lower than 20%, while other agents were less active *in vitro*. Infections caused by ECC with extensive carbapenem resistance herald a poor prognosis and have limited treatment options. Clonal spread of *bla*_NDM-1_ harboring *E. hormaechei* subsp. *xiangfangensis* ST171 and *bla*_VIM-1_ harboring *E. hormaechei* subsp. *hoffmannii* ST78, which have not been reported in Taiwan, was responsible for the increased incidence of ECC with complete resistance to tested carbapenems since 2019.

**IMPORTANCE:**

This study reported the increase of carbapenemase-producing *Enterobacter cloacae* complex (ECC) isolates at a medical center in southern Taiwan during 2014 and 2022. The emergence of NDM-1-producing *E. hormaechei* subsp. *xiangfangensis* ST171 and VIM-1-producing *E. hormaechei* subsp. *hoffmannii* ST78, contrary to widely reported IMP-8-producing carbapenem-resistant *E. cloacae* complex, accounts for the increased incidence of health care-associated infections with few remaining treatment options and poor clinical outcomes. Our findings highlighted the changing antimicrobial resistance of carbapenem resistance among Enterobacterales, leading to clinical difficulties in Taiwan.

## INTRODUCTION

*Enterobacter cloacae* complex (ECC) is among the list of important pathogens causing hospital-acquired infections, including bloodstream infections, urinary tract infections, and ventilator-associated or hospital-acquired pneumonia around the world (1–3). The emergence of antimicrobial resistance leads to treatment difficulties and patient morbidities, along with other well-known multidrug-resistant microbes, such as *Klebsiella pneumoniae* or *Acinetobacter baumannii* (4, 5). ECC isolates are able to produce beta-lactamases, including chromosomal AmpC beta-lactamases, extended spectrum beta-lactamases (ESBL), and carbapenemases (6). Moreover, antimicrobial resistance can be caused by modifications of membrane proteins, porins, efflux pumps, or mutation in antibiotic targets (6).

Carbapenems are the recommended agents for severe ECC infections, though cefepime or other non-beta-lactam agents are acceptable alternative choices (6, 7). For patients with ECC infections, the presence of carbapenem resistance decreases the likelihood of receiving appropriate antibiotics in time, which is detrimental for critically ill patients with septic shock (8). In the United States, the prevalence of carbapenem-resistant ECC (CR-ECC), which is defined as resistant to at least one carbapenem, increased from less than 1% in 2006 to 2.5% in 2015 (9). Production of carbapenemase, including IMP, IMI, KPC, NDM, NMC, OXA-48, VIM, FRI, and GES, became the main cause of carbapenem resistance among CR-ECC isolates worldwide since the late 2000s (10). The prevalence of ertapenem-resistant ECC in Taiwan ranged from 14.9% to 24.7% (11, 12). Among carbapenem-nonsusceptible ECC isolates obtained from 2016 to 2018, 41.4% can produce a carbapenemase (11). Of the 135 reported 135 carbapenemase-producing ECC isolates in Taiwan, 133 were able to produce IMP-8 (12–16), along with one isolate producing VIM-2 and another IMI-1 (14, 15).

Based on regular antimicrobial resistance surveillance at the study hospital, the incidence of ECC isolates resistant to three commonly used carbapenems, including ertapenem, imipenem, and meropenem, had increased since 2019. Eight of nine carbapenem-resistant isolates during 2021 and 2022 showed *in vitro* resistance to ceftazidime-avibactam using the gradient diffusion method, resulting in limited therapeutic options. The current study aimed to characterize the infection types, species identification, multilocus sequence typing (MLST), antimicrobial susceptibilities, and genes encoding beta-lactamases among ECC isolates showing resistance to all the tested carbapenems.

## MATERIALS AND METHODS

### Isolates and clinical information

During 2014 and 2022, clinical isolates of ECC showing resistance to all tested carbapenems including ertapenem, imipenem, and meropenem, using the disc diffusion method or Vitek 2 Gram-negative susceptibility card AST-N322 (bioMérieux, Durham, North Carolina, USA), were collected from the microbiology laboratory at National Cheng Kung University Hospital, a tertiary hospital with 1,354 beds located in southern Taiwan. If one patient had more than one isolate, only one isolate underwent further investigation. The study was approved by the Institutional Review Board of National Cheng Kung University Hospital (IRB No.: B-ER-112-082). The board waived the requirement of informed consent. The research was conducted in accordance with the Declaration of Helsinki, 2013 version, and institutional standards.

Clinical information, including demographic data, chronic diseases, risk factors for harboring multidrug resistance, sites and classifications of infection, vasopressor use, presence of septic shock, respiratory support, sequential organ failure assessment score (SOFA score) on the collection day, antibiotic regimens, treatment duration, and mortality (17), was obtained from the electronic health record. Septic shock was defined as vasopressor use plus a serum lactate ≥2 mM (18). ECC associated with colonization, defined by isolation of ECC but lack of clinical symptom, sign, or radiological findings supporting real infection, was excluded from further investigation. Healthcare-associated infections were defined by infections manifesting 48 h after admission to a hospital or healthcare facility.

### Microbiological testing

The ECC isolates were initially identified by matrix-assisted laser desorption ionization-time of flight mass spectrometry using VITEK MS system (bioMérieux, Lyon, France). The collected ECC isolates were grown in trypticase soy agar (TSA). The boiling method was used to extract DNA (19). The pellet was diluted in 0.5 mL of distilled water and boiled for 10 min to release DNA. The extracted DNA was subjected to polymerase chain reaction (PCR) and sequenced for multilocus sequence typing (MLST) by seven housekeeping genes (*dnaA*, *fusA*, *gyrB*, *leuS*, *pyrG*, *rplB*, and *rpoB*), as described by Miyoshi-Akiyama et al. (20). Determination of species and subspecies was performed according to the *hsp60* sequences (21). To detect the beta-lactamase, PCR was performed to target the genes encoding AmpC β-lactamase (*bla*_CMY_, *bla*_ACT_, and *bla*_DHA_) (22), ESBL (*bla*_SHV_, *bla*_CTX-M_, and *bla*_TEM_) (23), and carbapenemase (*bla*_KPC_, *bla*_IMI_, *bla*_IMP_, *bla*_VIM_, *bla*_NDM_, and *bla*_OXA-48_) (24).

Minimal inhibitory concentrations (MIC) were measured by the broth microdilution method using Vitek 2 Gram-negative susceptibility card AST-N322 and AST-XN09. The two AST cards had the same amikacin and meropenem sets but different tigecycline concentrations (0.75, 2, and 4 µg/mL vs 1.5, 4, and 8 µg/mL) on its plates. The susceptibility results were interpreted according to the CLSI M100 32nd ed document (25). For the agents with no clinical breakpoints available in the CLSI M100, the EUCAST breakpoints version 12.0 was used (26).

### Whole genome analysis and construction of phylogenetic tree

Whole-genome sequencing (WGS) was performed on selected *Enterobacter cloacae* complex strains representing commonly isolated sequence types. Sequencing was carried out using the PacBio Sequel IIe system (Pacific Biosciences Inc., Menlo Park, CA, USA). Genomic DNA was extracted and purified using the MagAttract HMW DNA Kit (Qiagen, Germany). Library preparation and sequencing were performed according to the manufacturer’s protocol (PN: 101-696-100, version 8). Raw sequencing data were processed and assembled using the SMRT Link software version 11 (Pacific Biosciences Inc., Menlo Park, CA, USA). The assembled genomes were submitted to the NCBI database under BioProject accession number PRJNA1248189. All sequence data could be accessed at the NCBI BioSample database.

Phylogenomic analysis of the sequenced isolates was conducted using the randomized accelerated maximum likelihood (RAxML) method, implemented through the Bacterial and Viral Bioinformatics Resource Center (https://www.bv-brc.org/). The resulting phylogenetic trees were visualized using the Molecular Evolutionary Genetics Analysis (MEGA) software (https://www.megasoftware.net/).

### Statistical analysis

All statistical analyses were performed using R 4.4.1 (R Core Team, http://www.R-project.org/, Vienna, Austria). Continuous variables were presented as means ± standard deviations. Wilcoxon rank-sum test was used for the between-group comparisons on numeric variables. Between-group in-hospital mortality was compared by Fisher’s exact test. Two-tail *P*-values of less than 0.05 were considered to be statistically significant.

## RESULTS

Among ECC isolates resistant to all tested carbapenems during 2014 and 2022 obtained from 118 patients, 68 isolates of ECC with resistance to ertapenem, imipenem, and meropenem from 64 patients were collected, while other isolates were not available due to logistic or preservation issues. Fifty-four isolates caused clinical infections during 2015 and 2022, while 10 were considered colonizers. Thirty-three (61.1%) of the 54 isolates were collected during their intensive care unit (ICU) stay. The demographic data, chronic diseases, and conditions associated with immunosuppression or presence of multi-drug resistant organisms are presented in Table 1. The mean age is 67.1 years. Chronic diseases with a prevalence rate of >20% include hypertension, solid organ malignancies, diabetes mellitus, chronic kidney disease, and heart failure. Recent medical interventions, such as mechanical ventilation (51.9%), surgical intervention (48.1%), and renal replacement therapy (35.2%), were not uncommon. Approximately 95% of patients received antibiotics within 90 days before the isolation of ECC, and half of all patients had carbapenem exposure.

**TABLE 1:** Characteristic of 54 patients with *Enterobacter cloacae* complex infection in a medical center in southern Taiwan^*b*^

Clinical variables	Numbers (%)
Age	67.1 ± 16.0^*a*^
Male sex	34 (63.0)
Chronic diseases	
Hypertension	27 (50.0)
Solid organ malignancy	21 (38.9)
Diabetes mellitus	19 (35.2)
Chronic kidney disease	15 (27.8)
Heart failure	14 (25.9)
Coronary artery disease	6 (11.1)
Old stroke	5 (9.3)
Cirrhosis	4 (7.4)
Chronic obstructive pulmonary disease or asthma	3 (5.6)
Hematologic malignancy	2 (3.7)
Connective tissue disease	2 (3.7)
Solid organ transplantation	2 (3.7)
Conditions associated with immunosuppression or MDRO	
Antibiotic exposure in the past 90 days	51 (94.4)
Carbapenem exposure	28 (51.9)
Mechanical ventilation	28 (51.9)
Recent operation	26 (48.1)
Renal replacement therapy	19 (35.2)
Immunosuppressant use	10 (18.5)
Ongoing chemotherapy	9 (16.7)
Corticosteroid use	6 (11.1)
Neutropenia	5 (9.3)

^*a*^ Mean ± standard deviation.

^*b*^ MDRO, multi-drug resistant organisms.

The distribution of infection types, clinical severities, antibiotic treatment, and mortality rates is shown in Table 2. Among 54 ECC infections, 52 (96.3%) were healthcare-associated infections. Major infections caused by ECC included bloodstream infections (20, 37.0%), lower respiratory tract infections (14, 25.9%), and surgical site infections (7, 13.0%). Septic shock occurred in 19 (29.7%) patients, and 29 patients (53.7%) needed invasive mechanical ventilation. Fifty-two percent of those with ECC infection died in the hospital. Among patients with septic shock, 78.9% did not survive to discharge. Commonly prescribed antibiotics included colistin, amikacin, and ciprofloxacin, while 14 (25.9%) patients did not receive any *in vitro* active agent. Patients without effective antibiotic therapy did not have a statistically higher in-hospital mortality rate than those receiving effective therapy (71.4%, 10/14 vs 45.0%, 18/40, *P* = 0.1235).

**TABLE 2:** Distribution of infection types, severities, antibiotic treatment, and mortality rates in 54 patients with infections due to carbapenem-resistant *E. cloacae* complex

	Case number (%) *n* = 54
Hospital-acquired infections	52 (96.3)
Intensive care unit-onset infections	33 (61.1)
Infection type	
Bloodstream infection	20
Catheter-associat bloodstreamam infection	18
Lower respiratory tract infection	14
Ventilator-associated pneumonia	8
Community-acquired pneumonia	2
Hospital-acquired pneumonia	2
Tracheobronchitis	2
Surgical site infection (SSI)	7
Superficial SSI	2
Deep SSI	4
Organ / space SSI	1
Urinary tract infection	6
Biliary tract infection	4
Intra-abdominal infection	2
Skin and soft tissue infectionn	1
Disease severities	
Mechanical ventilation	29 (53.7)
Vasopressor use	26 (48.1)
Septic shock	19 (35.1)
SOFA score	8.1 ± 5.6^*a*^
Antibiotic treatment	
Colistin	18 (33.3)
Amikacin	13 (24.1)
Ciprofloxacin	12 (22.2)
Other agents^*b*^	1-3 each
No *in vitro* active drug	14 (25.9)
28-day mortality	26 (48.1)
In-hospital mortality	28 (51.8)

^*a*^ Mean ± standard deviation.

^*b*^ Other agents included: tigecycline (3), gentamicin (3), levofloxacin (2), meropenem (1), piperacillin-tazobactam (1), cefepime (1), ceftazidime-avibactam (1).

**Fig 1:**
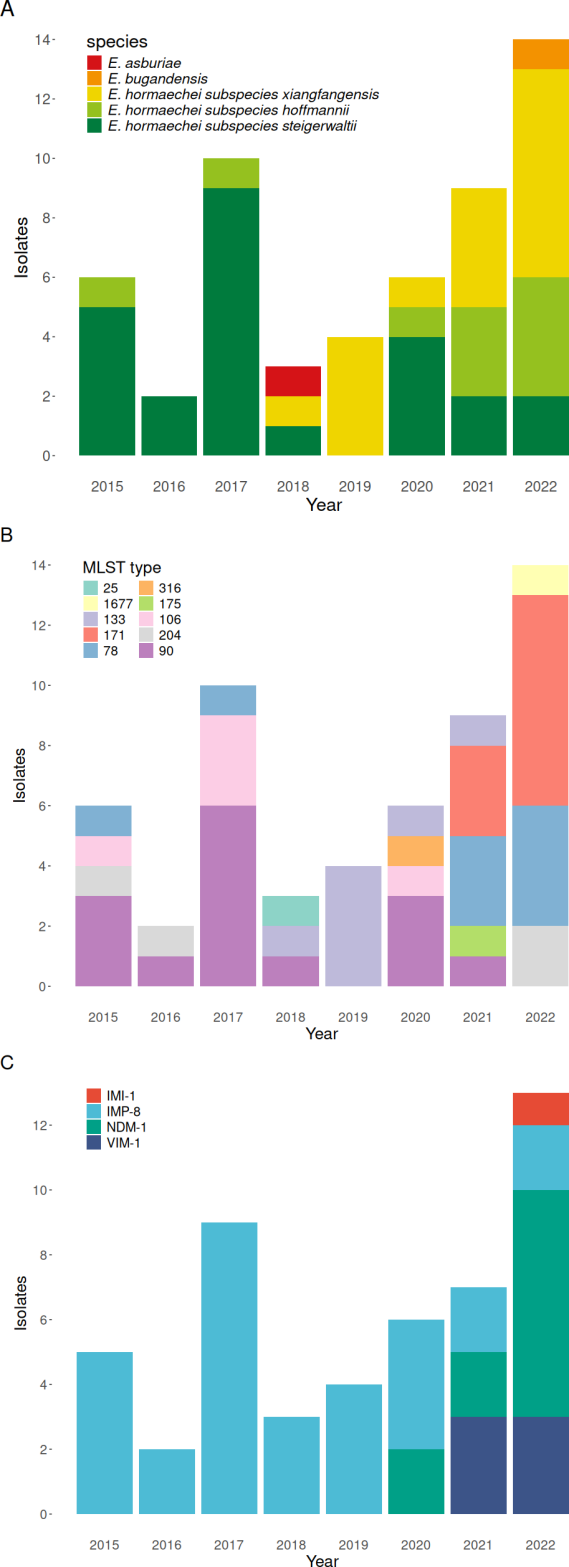
Distribution of (**A**) species, (**B**) multilocus sequence typing (MLST) types, and (**C**) carbapenemase genes among the 54 carbapenem-resistant *E. cloacae* complex isolates during 2015 and 2022.

The distribution of species and MLST types is shown in Table 3; Fig. 1A and B. The vast majority of ECC isolates belonged to *Enterobacter hormaechei* (52, 96.3%), which can be classified as three subspecies. *E. hormaechei* subsp. *steigerwaltii* was the most common, accounting for 46.3% (25 isolates) of the tested ECC isolates (Table 3). As for *E. hormaechei* subsp. *xiangfangensis*, a total of 17 isolates were identified, and it was first noted since 2018 and predominant in 2021 and 2022 due to the emergence of ST171. The third subspecies of *E. hormaechei* was *E. hormaechei* subsp. *hoffmannii* (10 isolates), mainly ST78 found in 2021 and 2022 (Fig. 1B). The other two species were *E. asburiae* (one isolate) and *E. bugandensis* (1).

**TABLE 3:** Species, multilocus sequence typing (MLST) types, carbapenemase and extended-spectrum beta-lactamase (ESBL)-producing genes among *E. cloacae* complex isolates

Species and subspecies	MLST (isolate number)	Isolates with beta-lactamase encoding genes^*c*^					
		*bla*_IMP-8_	*bla*_NDM-1_	*bla*_VIM-1_	*bla*_IMI-1_	*bla*_SHV-12_	*bla*_CTX-M_
*E. hormaechei*							
subsp. *steigerwaltii*	90 (15)	13	1			12	1^*a*^
	106 (5)	5				5	
	204 (4)	3				3	
	175 (1)						1^*b*^
subsp. *xiangfangensis*	171 (10)		9				3^*b*^
	133 (7)	7					
subsp. *hoffmannii*	78 (9)	2		6		6	1^*a*^
	316 (1)		1				
*E. asburiae*	25 (1)	1					1^*b*^
*E. bugandensis*	1677 (1)				1		

^*a*^ CTX-M-3.

^*b*^ CTX-M-15.

^*c*^ The empyt cells mean "0".

Of 54 ECC isolates with complete resistance to the tested carbapenems, 49 (90.7%) harbored carbapenemase-encoding genes, including *bla*_IMP-8_ (31 isolates), *bla*_NDM-1_ (11), *bla*_VIM-1_ (6), and *bla*_IMI-1_ (1). Among MBL-encoding genes, *bla*_NDM-1_ was detected since 2019 and became the major carbapenemase-encoding gene in 2022 (7/13, 53.8%; Fig. 1C). *bla*_NDM-1_ was mainly found in *E. hormaechei* subsp. *xiangfangensis* (ST171, nine isolates), while *bla*_VIM-1_ was constantly associated with *E. hormaechei* subsp. *hoffmannii* ST78. Twenty-eight (57.1%) of 49 isolates with MBL-encoding genes concurrently harbored ESBL-encoding genes. All five ECC isolates without MBL-encoding genes possessed ESBL-encoding genes, including *bla*_SHV-12_ (two isolates), *bla*_CTX-M-15_ (two isolates), *bla*_CTX-M-3_ (one isolate), in addition to the gene encoding chromosomal AmpC beta-lactamase (four isolates with *bla*_ACT_ and one isolate with both *bla*_CMY-6_ and *bla*_ACT_). Of note, no isolate with the genes encoding KPC or OXA-48 was detected.

The MIC results are demonstrated in Table 4. Nearly all isolates were resistant to cefotaxime and ceftolozane-tazobactam. Less than 10% were susceptible to cefepime or piperacillin-tazobactam. The carbapenem resistance was higher than 85%, and all isolates were resistant to at least one carbapenem. Eight isolates not demonstrating ertapenem resistance had their ertapenem susceptibility results masked by the Vitek 2 AST system, as they had high imipenem and meropenem MIC values higher than 16 µg/mL. All these eight isolates carried *bla*_IMP-8_. There was another isolate with *bla*_IMP-8_ showing intermediate susceptibility to imipenem and resistant to ertapenem and meropenem. Additionally, two isolates without carbapenemase-producing genes showed resistance to ertapenem but variable susceptibility to imipenem and meropenem.

**TABLE 4:** Minimal inhibitory concentrations (MICs) and susceptibility interpretation for 54 *E. cloacae* complex isolates^*c*^^,^^*d*^

	Vitek2 AST-N322			Vitek2 AST-XN90		
	μg/mL		S / I^*a*^ / R (%)	μg/mL		S / I/ R (%)
	MIC_50_	MIC_90_		MIC_50_	MIC_90_	
Cefotaxime	≥64	≥64	0/0/100	≥64	≥64	0/0/100
Ceftazidime	≥64	≥64	0/0/100			
Cefepime	16	≥64	7.4/31.5/61.1			
Piperacillin-tazobactam	≥128	≥128	3.7/3.7/92.6			
Aztreonam				≥64	≥64	33.3/0/66.7
Ceftolozane-tazobactam				≥32	≥32	0/0/100
Ceftazidime-avibactam				≥16	≥16	11.1/0/88.9
Ertapenem	≥8	≥8	14.8/0/85.2			
Imipenem	≥16	≥16	1.9/3.7/94.4			
Meropenem	≥16	≥16	1.9/0/98.1	≥16	≥16	1.9/0/98.1
Levofloxacin	≥8	≥8	7.4/16.7/75.9			
Ciprofloxacin	>4	>4	20.3/1.9/77.8			
Moxifloxacin^*b*^				≥8	≥8	3.7/0/96.3
Gentamicin	≥16	≥16	24.1/13.0/63.0			
Amikacin	≤2	16	83.3/1.9/14.8	≤2	16	83.3/3.7/13.0
Colistin	≤0.5	≤0.5	0/100/0			
Tetracycline				≥16	≥16	31.5/3.7/64.8
Tigecycline^*b*^	4	≥8	3.7/0/96.3	2	≥8	24.1/0/75.9
TMP/SMX	≥16	≥16	20.4/0/79.6			

^*a*^ Including intermediate and susceptible-dose-dependent categories.

^*b*^ Interpretation according to the EUCAST clinical breakpoints.

^*c*^ TMP/SMX: trimethoprim-sulfamethoxazole.

^*d*^ The empty cells mean that the AST card did not have the drug on its panel so no testing result is available.

One third of the isolates were susceptible to aztreonam, and 91.7% of these resistant isolates harbored either *bla*_SHV-12_ or *bla*_CTX-M_. Notably, among all the tested ECC isolates, 88.9% exhibited resistance to ceftazidime-avibactam, and all of these isolates possessed at least one MBL-producing gene. In contrast, five of the six ceftazidime-avibactam-susceptible isolates lacked a carbapenemase-encoding gene, and one carried only the non-MBL carbapenemase gene *bla*_IMI-1_. Among the other antimicrobial agents, only amikacin and colistin had resistance rates of <20%, while ciprofloxacin, levofloxacin, gentamicin, tobramycin, tetracycline, tigecycline, and trimethoprim-sulfamethoxazole showed susceptibility rates of <50%. Among the 13 isolates having susceptibility to tigecycline (MIC ≤0.5 µg/mL) tested by AST-XN09 cards, only two had the same results using AST-N322 cards, while four isolates had MIC values of 1 µg/mL, and seven had MIC values of 2 µg/mL.

**Fig 2:**
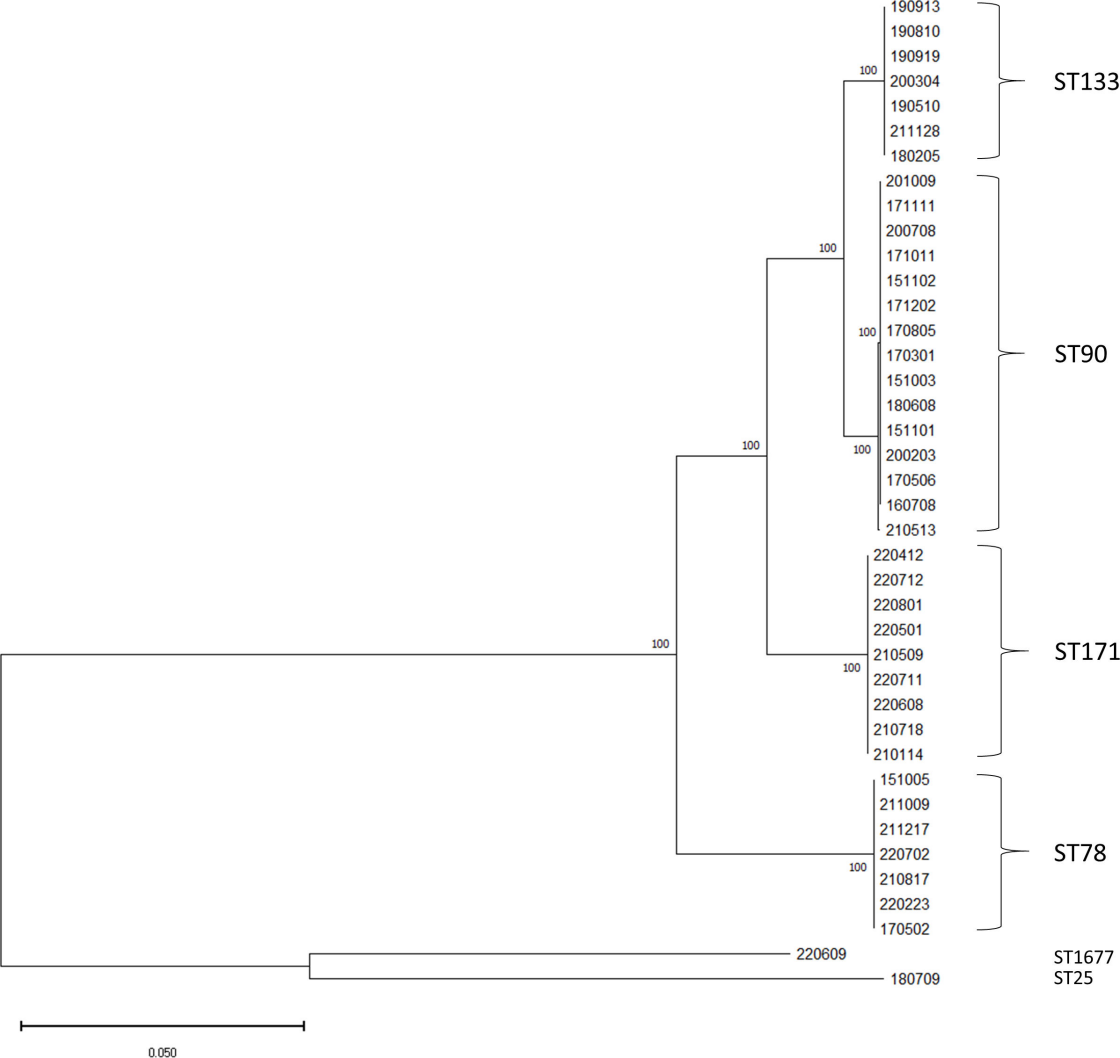
Phylogenetic tree of the 40 selected strains, including four commonly isolated sequence types (ST78, ST90, ST133, and ST171) of *E. hormaechei, one E. asburiae* (ST25) *and E. bugandensis* (ST1677). The scale bar indicates a sequence divergence of 5%.

The phylogenomic analysis of the sequenced isolates is shown in Fig. 2. Four clusters of isolates corresponded to E. *hormaechei* ST78, 90, 133, and 171, with considerable genetic distance between these clustered isolates and other *E. cloacae* complex such as *E. asburiae* and *E. bugandensis*.

## DISCUSSION

The species identification, MLST, beta-lactamase detection, and WGS analysis provide new insights into the changing epidemiology of infections caused by the ECC isolates with complete resistance to the three carbapenems at the study hospital. The MLST and WGS analysis supported the clonal spread of *E. hormaechei* ST78, ST133, and ST171 at the study hospital. NDM-1, not reported among ECC isolates in Taiwan, has accounted for the majority of carbapenem-resistant isolates since 2019. The emergence of *E. hormaechei* subsp. *xiangfangensis* ST171 in 2021 led to the surge of clinical ECC isolates resistant to all the tested carbapenems. Contrary to the KPC-producing carbapenem-resistant ST171 in the United States (10, 27–29), nine of ten ST171 isolates in this study carried *bla*_NDM-1_. NDM-1 producing *E. hormaechei* subsp. *xiangfangensis* ST171 isolates had been reported from Vietnam (30), and also South Africa, Guatemala, India, and China (31–34). NDM-5, another NDM subtype frequently reported, was more prevalent than NDM-1 in some studies, among ST171 isolates in China (33, 35–38). Moreover, ST171 isolates from Spain and Nepal could produce OXA-48 carbapenemase, as well (39, 40). None of our isolates harbored *bla*_KPC_ or *bla*_OXA-48_, emphasizing the global diversity of carbapenemase distribution and the need for regional genotypic surveillance of the mechanism of carbapenem resistance.

The *bla*_VIM-1_-harboring *E. hormaechei* subsp. *hoffmannii* ST78 contributed to the increased occurrence of ECC isolates resistant to the three tested carbapenems in the current study. Although *bla*_VIM-1_ has not been previously reported in any ECC isolate from Taiwan, ST78 can carry a variety of carbapenemase genes, including *bla*_KPC-3_, *bla*_KPC-4_, *bla*_IMP-4_, *bla*_IMP-8_, *bla*_OXA-48_, *bla*_VIM-1_, and *bla*_IMI-1_ in North America, Europe, and Japan (27, 31, 41, 42). On the other hand, *bla*_IMP-8_ which had been noted in Taiwan since the 1990s (12, 14, 16, 43), remained the most common carbapenemase genes in this study. The annual number of *bla*_IMP-8_ harboring ECC isolates ranged from two to nine in the current study, and *bla*_IMP-8_ was present among three subspecies of *E. hormaechei* (subsp. *steigerwaltii, hoffmannii,* and *xiangfangensis*), and *E. asburiae*.

In the clinical aspect, infections caused by these carbapenem-resistant ECC were associated with an in-hospital mortality rate of higher than 50%, possibly related to inability to receive effective therapy in time. The susceptibility data indicated *in vitro* resistance to many antimicrobial agents, including aztreonam, ceftazidime-avibactam, fluoroquinolones, trimethoprim-sulfamethoxazole, and tigecycline. Physicians are forced to empirically administer nephrotoxic agents, including amikacin or colistin, for patients with suspected infections caused by ECC with extensive carbapenem resistance. Cefiderocol or the combination of aztreonam and ceftazidime-avibactam, if available, could be considered for managing difficult-to-treat CR-ECC infections (44, 45).

The current study has several limitations. First, the findings of a single center may not be extrapolated to other settings. The retrospective design may underestimate the real incidence of CR-ECC infections. Second, a higher prevalence of carbapenemase-encoding genes among our CR-ECC isolates may be related to the strict inclusion criterion of *in vitro* resistance to all three carbapenems, since ertapenem mono-resistant Enterobacterales has been associated with a low carriage rate (2.4%) of carbapenemase genes (46). Some carbapenemase-producing Enterobacterales, such as OXA-48 producers, might not be included as they present variable susceptibility to meropenem or imipenem (47). Third, the relatively small case number may be underpowered to demonstrate the between-group differences. Finally, the carbapenem resistance phenotype might not be exactly predicted by the carbapenemase genotype, since carbapenem resistance can be mediated by diverse mechanisms alone or in combination, in addition to the presence of carbapenemase genes, among CR-ECC isolates (6).

The distribution of species, MLST, and carbapenemase genes of clinical ECC isolates evolved in southern Taiwan during 2014 and 2022. The emergence of NDM-1-producing *E. hormaechei* subsp. *xiangfangensis* ST171 and VIM-1-producing *E. hormaechei* subsp. *hoffmannii* ST78 is responsible for the increasing prevalence of ECC with extensive carbapenem resistance, which heralds grave clinical outcomes, at least partially due to limited treatment options. Multi-center surveillance studies are warranted to comprehensively address the extent of carbapenem resistance and feasible preventive interventions among ECC isolates in Taiwan.

## SUPPLEMENTAL MATERIAL

Table S1 spectrum.00234-25-s0001.docx Access to sequence data of selected ECC isolates.

ASM does not own the copyrights to Supplemental Material that may be linked to, or accessed through, an article. The authors have granted ASM a non-exclusive, world-wide license to publish the Supplemental Material files. Please contact the corresponding author directly for reuse.
