# Supplementary material for: Infections caused by clonal spread of metallo-beta-lactamase-producing Enterobacter cloacae complex isolates at a southern Taiwan hospital
Source: Microbiol Spectr. 2025 Jun 10;13(7):e00234-25. doi: 10.1128/spectrum.00234-25 (PMC12210983; doi:10.1128/spectrum.00234-25)
Supplement: Table S1 — Access to sequence data of selected ECC isolates. [file spectrum.00234-25-s0001.docx]

Table S1. The sequence data for the 40 *Enterobacter cloacae complex* isolates has been deposited in the NCBI BioSample database. The corresponding accession numbers, sample names, and website links are summarized.

| BioSample | Sample name | Strain | Organism | Website |
| --- | --- | --- | --- | --- |
| SAMN47852679 | *Enterobacter hormaechei* 108 | 151005 | *Enterobacter hormaechei* | https://www.ncbi.nlm.nih.gov/biosample/47852679 |
| SAMN47852680 | *Enterobacter hormaechei* 109 | 151003 | *Enterobacter hormaechei* | https://www.ncbi.nlm.nih.gov/biosample/47852680 |
| SAMN47852681 | *Enterobacter hormaechei* 160 | 151101 | *Enterobacter hormaechei* | https://www.ncbi.nlm.nih.gov/biosample/47852681 |
| SAMN47852682 | *Enterobacter hormaechei* 110 | 151102 | *Enterobacter hormaechei* | https://www.ncbi.nlm.nih.gov/biosample/47852682 |
| SAMN47852683 | *Enterobacter hormaechei* 113 | 160708 | *Enterobacter hormaechei* | https://www.ncbi.nlm.nih.gov/biosample/47852683 |
| SAMN47852684 | *Enterobacter hormaechei* 161 | 170301 | *Enterobacter hormaechei* | https://www.ncbi.nlm.nih.gov/biosample/47852684 |
| SAMN47852685 | *Enterobacter hormaechei* 116 | 170502 | *Enterobacter hormaechei* | https://www.ncbi.nlm.nih.gov/biosample/47852685 |
| SAMN47852686 | *Enterobacter hormaechei* 117 | 170506 | *Enterobacter hormaechei* | https://www.ncbi.nlm.nih.gov/biosample/47852686 |
| SAMN47852687 | *Enterobacter hormaechei* 118 | 170805 | *Enterobacter hormaechei* | https://www.ncbi.nlm.nih.gov/biosample/47852687 |
| SAMN47852688 | *Enterobacter hormaechei* 119 | 171011 | *Enterobacter hormaechei* | https://www.ncbi.nlm.nih.gov/biosample/47852688 |
| SAMN47852689 | *Enterobacter hormaechei* 120 | 171111 | *Enterobacter hormaechei* | https://www.ncbi.nlm.nih.gov/biosample/47852689 |
| SAMN47852690 | *Enterobacter hormaechei* 122 | 171202 | *Enterobacter hormaechei* | https://www.ncbi.nlm.nih.gov/biosample/47852690 |
| SAMN47852691 | *Enterobacter hormaechei* 123 | 180205 | *Enterobacter hormaechei* | https://www.ncbi.nlm.nih.gov/biosample/47852691 |
| SAMN47852692 | *Enterobacter hormaechei* 158 | 180608 | *Enterobacter hormaechei* | https://www.ncbi.nlm.nih.gov/biosample/47852692 |
| SAMN47852693 | *Enterobacter asburiae* 159 | 180709 | *Enterobacter asburiae* | https://www.ncbi.nlm.nih.gov/biosample/47852693 |
| SAMN47852694 | *Enterobacter hormaechei* 137 | 190510 | *Enterobacter hormaechei* | https://www.ncbi.nlm.nih.gov/biosample/47852694 |
| SAMN47852695 | *Enterobacter hormaechei* 138 | 190810 | *Enterobacter hormaechei* | https://www.ncbi.nlm.nih.gov/biosample/47852695 |
| SAMN47852696 | *Enterobacter hormaechei* 139 | 190913 | *Enterobacter hormaechei* | https://www.ncbi.nlm.nih.gov/biosample/47852696 |
| SAMN47852697 | *Enterobacter hormaechei* 140 | 190919 | *Enterobacter hormaechei* | https://www.ncbi.nlm.nih.gov/biosample/47852697 |
| SAMN47852698 | *Enterobacter hormaechei* 129 | 200203 | *Enterobacter hormaechei* | https://www.ncbi.nlm.nih.gov/biosample/47852698 |
| SAMN47852699 | *Enterobacter hormaechei* 127 | 200304 | *Enterobacter hormaechei* | https://www.ncbi.nlm.nih.gov/biosample/47852699 |
| SAMN47852700 | *Enterobacter hormaechei* 209 | 200708 | *Enterobacter hormaechei* | https://www.ncbi.nlm.nih.gov/biosample/47852700 |
| SAMN47852701 | *Enterobacter hormaechei* 163 | 201009 | *Enterobacter hormaechei* | https://www.ncbi.nlm.nih.gov/biosample/47852701 |
| SAMN47852702 | *Enterobacter hormaechei* 141 | 210114 | *Enterobacter hormaechei* | https://www.ncbi.nlm.nih.gov/biosample/47852702 |
| SAMN47852703 | *Enterobacter hormaechei* 142 | 210509 | *Enterobacter hormaechei* | https://www.ncbi.nlm.nih.gov/biosample/47852703 |
| SAMN47852704 | *Enterobacter hormaechei* 131 | 210513 | *Enterobacter hormaechei* | https://www.ncbi.nlm.nih.gov/biosample/47852704 |
| SAMN47852705 | *Enterobacter hormaechei* 132 | 210718 | *Enterobacter hormaechei* | https://www.ncbi.nlm.nih.gov/biosample/47852705 |
| SAMN47852706 | *Enterobacter hormaechei* 133 | 210817 | *Enterobacter hormaechei* | https://www.ncbi.nlm.nih.gov/biosample/47852706 |
| SAMN47852707 | *Enterobacter hormaechei* 135 | 211009 | *Enterobacter hormaechei* | https://www.ncbi.nlm.nih.gov/biosample/47852707 |
| SAMN47852708 | *Enterobacter hormaechei* 136 | 211128 | *Enterobacter hormaechei* | https://www.ncbi.nlm.nih.gov/biosample/47852708 |
| SAMN47852709 | *Enterobacter hormaechei* 166 | 211217 | *Enterobacter hormaechei* | https://www.ncbi.nlm.nih.gov/biosample/47852709 |
| SAMN47852710 | *Enterobacter hormaechei* 144 | 220223 | *Enterobacter hormaechei* | https://www.ncbi.nlm.nih.gov/biosample/47852710 |
| SAMN47852711 | *Enterobacter hormaechei* 210 | 220412 | *Enterobacter hormaechei* | https://www.ncbi.nlm.nih.gov/biosample/47852711 |
| SAMN47852712 | *Enterobacter hormaechei* 148 | 220501 | *Enterobacter hormaechei* | https://www.ncbi.nlm.nih.gov/biosample/47852712 |
| SAMN47852713 | *Enterobacter hormaechei* 150 | 220608 | *Enterobacter hormaechei* | https://www.ncbi.nlm.nih.gov/biosample/47852713 |
| SAMN47852714 | *Enterobacter hormaechei* 168 | 220702 | *Enterobacter hormaechei* | https://www.ncbi.nlm.nih.gov/biosample/47852714 |
| SAMN47852715 | *Enterobacter bugandensis* 167 | 220609 | *Enterobacter bugandensis* | https://www.ncbi.nlm.nih.gov/biosample/47852715 |
| SAMN47852716 | *Enterobacter hormaechei* 152 | 220712 | *Enterobacter hormaechei* | https://www.ncbi.nlm.nih.gov/biosample/47852716 |
| SAMN47852717 | *Enterobacter hormaechei* 151 | 220711 | *Enterobacter hormaechei* | https://www.ncbi.nlm.nih.gov/biosample/47852717 |
| SAMN47852718 | *Enterobacter hormaechei* 153 | 220801 | *Enterobacter hormaechei* | https://www.ncbi.nlm.nih.gov/biosample/47852718 |
